# New advances and future directions in plant polyspermy

**DOI:** 10.1002/mrd.23261

**Published:** 2019-09-12

**Authors:** Dawit G. Tekleyohans, Rita Groß-Hardt

**Affiliations:** Centre for Biomolecular Interactions, University of Bremen, Bremen, Germany

**Keywords:** hybridization, plant, polyploidization, polyspermy

## Abstract

Plants have evolved a battery of mechanisms that potentially act as polyspermy barriers. Supernumerary sperm fusion to one egg cell has consequently long remained a hypothetical concept. The recent discovery that polyspermy in flowering plants is not lethal but generates viable triploid plants is a game changer affecting the field of developmental biology, evolution, and plant breeding. The establishment of protocols to artificially induce polyspermy together with the development of a high-throughput assay to identify and trace polyspermic events in planta now provide powerful tools to unravel mechanisms of polyspermy regulation. These achievements are likely to open new avenues for animal polyspermy research as well, where forward genetic approaches are hampered by the fatal outcome of supernumerary sperm fusion.

## 1 Background

Since the time of adaptive radiation of life on dry land, higher plants have evolved several traits that enable them to spread across the globe. Among the important achievements is a cellular sperm transportation system called pollen. In most sexually reproducing plants, it is this pollen which by growing out into a tubular structure delivers one pair of sperm cells that ultimately fertilize the two female gametes, egg and central cell. While the fertilized egg cell develops into the embryo, the fertilized central cell gives rise to the endosperm, which serves to nourish the developing embryo (Russel, 1992; Johnson et al., 2019). Unlike seaweeds and lower plants, where the egg cell is challenged with several motile sperm cells, often a 1:1 sperm to egg ration is kept in such double fertilization mode of reproduction. One likely parameter for this low sperm to egg ratio is the so-called pollen tube block. The power of this mechanism lies in coupling double fertilization to the disintegration of the pollen tube attracting machinery: In most plants, synergid cells within the ovule secret chemical cues to attract pollen tubes and assist the delivery of a pair of sperm cell that fertilize the two female gametes (Higashiyama, 2002; Higashiyama et al., 2001; Márton et al., 2005; Dresselhaus, 2005; Okuda et al., 2009). Sperm cell release and gamete fusion trigger sequential disintegration of both synergids such that typically only a single pollen tube is attracted to one ovule (Maruyama et al., 2015; Völz et al., 2013). In events of incomplete fertilization, when only one of the two female gametes gets fertilized, degeneration of the second synergid will not takes place; instead a second pollen tube is attracted to complete the fertilization process (Beale et al., 2012; Kasahara et al., 2012; Sprunck et al., 2012). To standardize nomenclature with research from the animal field, the terms “polyspermy” and “monospermy” on their own refer to the egg cell unless otherwise specified (e.g., central cell polyspermy).

Until recently, it was unclear whether polyspermy occurs in flowering plants and the closest organisms for which functional evidence was available were fucoid algae (Brawley, 1987). In these species, polyspermy is lethal with zygote development ceasing at the four-cell stage (Brawley, 1987). To circumvent the fatal consequences of supernumerary gamete fusion, several organisms have evolved polyspermy barriers. Such prevention mechanisms are multi phasic and can be grouped into distinct categories: Fast, intermediate, and slow block (Spielman & Scott, 2008). The fast polyspermy block is often manifested through a change in egg cell membrane potential within seconds after gamete fusion and is reported to be a sodium-dependent phenomenon in the case of fucoid algae (Brawley, 1987; Brawley, 1991). In animals, this electrical response is commonly linked to a permanent egg cell block through the release of Ca^2+^ from internal stores and the secretion of cortical granules, which induce a chemical renovation of the egg cell matrix. And there is evidence that similar mechanisms operate in plants (see below).

Removal of egg surface coat components essential for gamete recognition and fusion has been reported in several species as an intermediate means of polyspermy block (Bianchi & Wright, 2014; Brawley, 1990b; Tekleyohans et al., 2017; Vacquier et al.,1973). FUS1 and HAP2 proteins are two gamete-specific components essential for gamete fusion in Chlamydomonas (Ferris et al., 1996; Liu et al., 2008; Misamore et al., 2003). Importantly, these proteins become eliminated from the plasma membrane after gamete fusion, and this process correlates with a reduction in the fusogenic capacity of the gametic cell so as to prevent polygamy (Liu et al., 2010). Also in flowering plants, HAP2/GCS1 has been reported to be an essential sperm component required for gamete fusion (Mori et al., 2006; Valansi et al., 2017; von Besser et al., 2006). The corresponding egg cell plasma membrane localized factor that is essential for the gamete fusion is not yet known but we speculate that similar mechanisms might operate as well in flowering plants. Intriguingly, the presentation of HAP2/GCS1 on the sperm surface is under regulatory control by the egg cell, which transiently secrets EC1 protein thereby triggering the translocation of HAP2/GCS1 from the endomembrane system to the sperm coat (Sprunck et al., 2012). Whether this sperm activation by the egg constitutes contributes to monospermy is currently unknown.

Apart from the fast and intermediate response to gamete fusion, the egg is subjected to further modifications after fertilization. Based on in vitro studies conducted on rice, supernumerary sperm cell fusion is significantly prevented 10 min after the first sperm is fused with an egg cell (Toda et al, 2016). Such block to polyspermy could potentially be associated with the establishment of a protective cell wall after the gamete fusion, which according to in vitro maize gamete fusion study, can take up to 20min to shield the entire egg surface with cell wall material (Kranz et al., 1995). Secretion of cell wall material upon exposure of calcium ionophores has been reported in fucoid algae and maize egg cells suggesting that calcium mediates vesicle release into the egg matrix thereby establishing a permanent block to polyspermy (Antoine et al., 2000; Brawley, 1990a, 1991; Brawley & Bell, 1987; Kranz et al., 1995; Liu, 2011; Tsaadon et al., 2006). Interestingly, the two female gametes differ with respect to fertilization induced calcium dynamics with only the egg cell exhibting a transient calcium spike (Denninger et al., 2014; Hamamura et al., 2014). While speculative at this point, it is conceivable that the transient calcium spike within the egg cell is connected to the induction of a polyspermy block. In agreement with this, works on Arabidopsis thaliana and Zea mays have shown that the central cell has no, or a less strict block to polyspermy as compared with the egg cell (Grossniklaus, 2017; Scott et al., 2008).

## 2 Plant Polyspermy: Milestones and Challenges

Hardly any approach has shaped our understanding of developmental biological processes more than forward genetics. The random mutagenesis of genomes combined with often sophisticated screening techniques has lead with intriguing confidence to developmentally decisive loci. The fact that supernumerary sperm fusion has a lethal outcome in many organisms has prevented this technique from being used in the field of polyspermy. Notably, flowering plants exhibit a remarkable tolerance to genomic imbalance. In fact, crosses between tetraploid and diploid plants can yield viable triploid offspring (Scott et al., 1998), and it has therefore been questioned whether polyspermy has a lethal outcome (Spielman & Scott, 2008). Nevertheless, in the absence of tools that enable an unambiguous identification of polyspermic derived polyploids from other means of polyploidization mechanism, the fusion of two sperm cells with an egg cell in plants has long remained a hypothetical concept (Tekleyohans et al., 2017).

However, recent studies have made a significant advance in plant polyspermy research field by solving one of the main bottle neck, the detection and characterization of polyspermy-derived offspring. The first elegant study is capitalized on an in vitro fertilization technique where electric fusion is carried out between two sperm and one egg cell to form a triploid rice zygote, from which viable plants could be recovered (Toda et al., 2016). Such technique provides a powerful tool to study developmental profiles of polyspermic zygotes, embryos, and plants (reviewed by Toda et al. in this issue). To address naturally occurring polyspermy events, Nakel et al. (2017) took advantage of the GAL4/UAS two-component system adapted from yeast. Their high-throughput polypaternal breeding design comprises of one plant line containing the GAL4 transcription factor under a ubiquitous promoter and a second plant line containing the GAL4 responsive UAS enhancer driving a *YFP*-tagged herbicide resistance gene. The two lines serve both as pollen donors (fathers) for a third acceptor plant (mother). With this simplistic but straightforward genetic approach, the authors screened more than 120,000 seeds for herbicide resistance thereby identifying polyspermy-derived seedlings that were derived from three parents, one mother and two fathers. In fact, it was estimated that a single Arabidopsis plant gives rise to about five polyspermy-derived offspring under ideal growth conditions (Nakel et al., 2017).

**Figure 1:**
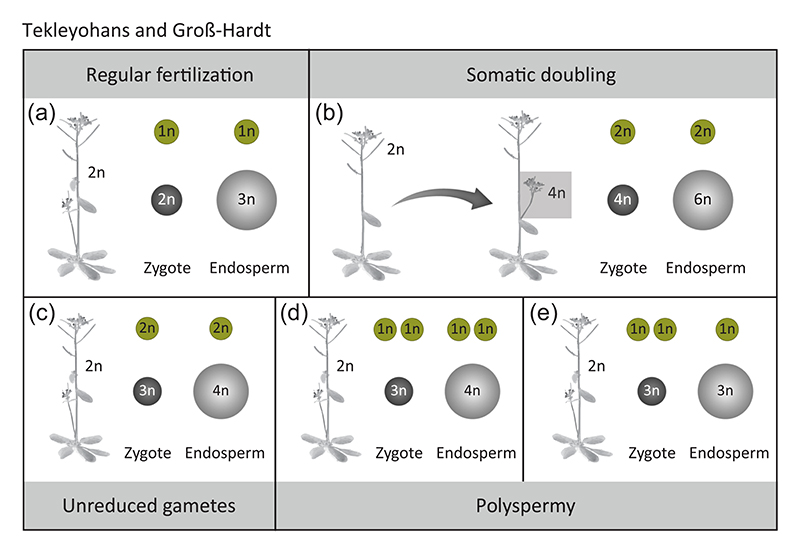
Plant polyploidization scenarios. (a) During regular fertilization, a haploid sperm (color) fertilizes a haploid egg and a diploid central cell to give rise to a diploid embryo and triploid endosperm, respectively. (b and c) Cell-cycle defects can result in somatic doubling (b) or unreduced gametes (c). Whilethe first scenario equally affects both sexes, unreduced gametes are considered mainlyto be formed in the male germline, resulting in an unbalanced female-male genome contribution in the seed. (d and e) It is currently unclear whether polyspermy introduces extra paternal copies into both gametes (d) or whether selective egg cell polyploidization is possible (e).

This finding has brought what used to be a concept into reality and improves the common understanding towards the origin of polyploids (Figure 1). Until now, it is assumed that polyploidization is mainly caused by cell-cycle defects, which can induce somatic doubling or the formation of unreduced gametes (Figure 1A-C). Particularly the formation of unreduced sperm has been considered a major route towards polyploid plants (Kreiner et al., 2017; Mason & Pires, 2015; Ramsey & Schemske, 1998). In fact, unreduced sperm have been observed in predominantly selfing Brassicaceae at a frequency of 1.8% (Kreiner et al., 2017). However due to the lack of tools to unambiguously trace the fate of unreduced gametes, the importance of this polyploidization scenario is difficult to assess.

## 3 Prospect

Understanding the origin and consequences of polyploidization is essential for several reasons. First, polyploid plants play an enormous role for plant breeding. In fact, most crop species cultivated today (e.g., coffee, wheat, sugar cane, maize, cotton, potato, rape seed, tomato, and apple) are polyploid, indicating that polyploidization, especially allopoly-ploidization contributed significantly to the productivity of these plants (Dar et al., 2017; Renny-Byfield & Wendel, 2014). Second, polyspermy not only facilitates polyploidization but also opens a new possibility to generate hybrids of three different genotypes within a single generation (Nakel et al., 2017), which is impossible to be attained by other means of reproduction. The integration of three different genomes in one generation not only induces hybrid vigor; it might, in addition, cause an unanticipated shock that results in genome restructuring and enhanced expression plasticity that potentially leads to the development of novel traits (Jackson & Chen, 2010). Third, polyspermy might have the potential to bypass hybridization barriers thereby facilitating the generation of new cultivars: Most canonical polyploidization scenarios involve the transmission of supernumerary genomic copies not only to the embryo but also to the endosperm (Figure 1a-c) instigating an interploidy hybridization barrier that result in seed abortion (Scott et al., 1998). While polyspermy induced seeds might similarly be subjected to this so-called triploid block (Figure 1d), it is theoretically conceivable that polyspermy selectively polyploidizes the egg cell only (Figure 1e). I will be an important task for the future to determine whether such a scenario occurs in planta.

The ability to artificially induce polyspermy by cell fusion and to trace naturally occurring polyspermy now allows to explore the evolutionary, developmental and breeding potential of this previously unrecognized reproductive mode. In addition, the survival of polyspermy-derived offspring makes polyspermy amendable to forward genetics and we anticipate that the results generated in the plant field will open up new avenues for animal polyspermy as well, where similar approaches are hampered by the lethal consequences of polyspermy.
